# Quantification of regional murine ozone-induced lung inflammation using [^18^F]F-FDG microPET/CT imaging

**DOI:** 10.1038/s41598-020-72832-8

**Published:** 2020-09-24

**Authors:** G. K. Aulakh, M. Kaur, V. Brown, S. Ekanayake, B. Khan, H. Fonge

**Affiliations:** 1 Western College of Veterinary Medicine, University of Saskatchewan, Saskatoon, Canada; 2 College of Medicine, University of Saskatchewan, Saskatoon, Canada; 3 Department of Medical Imaging, RUH Saskatoon, Saskatoon, Canada

**Keywords:** Physiology, Molecular medicine, Asthma, Respiratory distress syndrome, 3-D reconstruction, Molecular imaging, Positron-emission tomography, X-ray tomography

## Abstract

Ozone (O_3_) is a highly potent and reactive air pollutant. It has been linked to acute and chronic respiratory diseases in humans by inducing inflammation. Our studies have found evidence that 0.05 ppm of O_3_, within the threshold of air quality standards, is capable of inducing acute lung injury. This study was undertaken to examine O_3_-induced lung damage using [^18^F]F-FDG (2-deoxy-2-[^18^F]fluoro-D-glucose) microPET/CT in wild-type mice. [^18^F]F-FDG is a known PET tracer for inflammation. Sequential [^18^F]F-FDG microPET/CT was performed at baseline (i.e. before O_3_ exposure), immediately (0 h), at 24 h and at 28 h following 2 h of 0.05 ppm O_3_ exposure. The images were quantified to determine O_3_ induced spatial standard uptake ratio of [^18^F]F-FDG in relation to lung tissue density and compared with baseline values. Immediately after O_3_ exposure, we detected a 72.21 ± 0.79% increase in lung [^18^F]F-FDG uptake ratio when compared to baseline measures. At 24 h post-O_3_ exposure, the [^18^F]F-FDG uptake becomes highly variable (S.D. in [^18^F]F-FDG = 5.174 × 10^–4^ units) with a 42.54 ± 0.33% increase in lung [^18^F]F-FDG compared to baseline. At 28 h time-point, [^18^F]F-FDG uptake ratio was similar to baseline values. However, the pattern of [^18^F]F-FDG distribution varied and was interspersed with zones of minimal uptake. Our microPET/CT imaging protocol can quantify and identify atypical regional lung uptake of [^18^F]F-FDG to understand the lung response to O_3_ exposure.

## Introduction

Ozone (O_3_) is a toxic and highly reactive gaseous oxidizing chemical with well-documented adverse health effects in humans. On the basis of animal and human data, environmental guidelines and air quality standards recommend a threshold for exposure of no more than 0.063 ppm of O_3_ (average daily concentrations). Experiments done in animal models have shown that O_3_ induces acute lung injury, albeit at much higher and for longer O_3_ exposures (near 2 ppm for 3–6 h). Our research has standardized a sensitive model of sterile murine lung inflammation by exposing mice for two hours at 0.05 ppm O_3_, a level below the current recommendations for what is considered a safe or “ambient” O_3_ concentration for humans^1–3^. 0.05 ppm O_3_ exposure causes immediate lung neutrophil recruitment, release of IL-1β dependent cytokines in broncho-alveolar lavage and bone marrow mobilization of pan-leukocyte chemokine, SDF1α^2,3^. Thus, it is imperative to understand the progression of O_3_ induced early lung metabolic changes, at concentrations feasible in the environment.

[^18^F]F-FDG (2-deoxy-2-[^18^F]fluoro-D-glucose) microPET/CT imaging is a sensitive method to detect lung cancer and study various inflammatory diseases. The current quantification methods focus on tracer kinetics and compartment modelling such as the Patlak and Sokoloff methods^4,5^. However, the compartmental modelling does not convey the regional [^18^F]F-FDG activity pattern unless images are acquired through dynamic gating protocols and invasive blood sampling. Many pre-clinical microPET scanners resolve only up to 80 µm structures, which cannot resolve functional activity in the murine alveolar septa. Thus, we sought to fast-track the imaging protocol without invasive blood sampling. For understanding disease progression and regional tracer localization in relation to specific organs such as lungs, we developed an imaging protocol cum image analysis strategy to quantify the sequential uptake and distribution of [^18^F]F-FDG in murine lungs. We imaged each animal at four separate image time-points that spanned 2 days.

The resulting CT attenuation grey values were plotted against the percent (%) lung [^18^F]F-FDG SUR (standard uptake ratio). These plots provide quantifiable spatio-temporal lung FDG distribution patterns. Our results indicate that O_3_ initially induces higher and heterogeneous lung [^18^F]F-FDG uptake, when analyzed by X-ray CT guided region of interest (ROI) analysis of the PET image slices.

## Methods

### Mice

The study design was approved by the University of Saskatchewan’s Animal Research Ethics Board (AUP 20,170,016) and adhered to the Canadian Council on Animal Care guidelines for humane animal use. Six-eight week old male C57BL/6J (Stock No. 00064) mice were procured from Jackson Labs (CA, US).

### Ozone exposures

For O_3_ exposures, mice were continuously exposed in an induction box for 2 h^1,2^. These mice were housed in custom induction box and had free access to food and water. O_3_ (0.05 ± 0.02 ppm) was generated, at 3 L/min, from ultra-high-purity air using a silent-arc discharge O_3_ calibrator cum generator (2B Technologies, CO, USA). Constant chamber air temperature (72 ± 3°F) and relative humidity (50 ± 15%) were maintained. O_3_ concentrations were calibrated in small box using a real-time O_3_ monitor (2B Technologies, CO, USA).

### Experiment design

**Figure 1:**
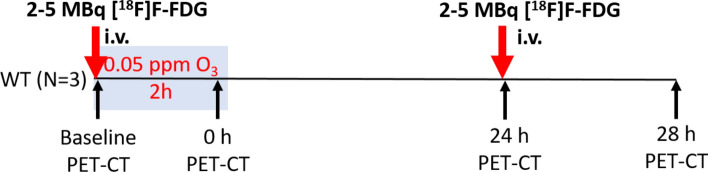
Experiment design and [^18^F]F-FDG PET-CT imaging protocol: Three pre-weighed fasted wild-type (WT) C57BL/6J mice were injected intravenously (i.v.) with 2–5 MBq of [^18^F]F-FDG. Each mouse was then prepared for isoflurane anaesthesia and imaged. After baseline imaging, mice were exposed to 0.05 ppm of ozone (O_3_) for 2 h. After exposure i.e. O_3_-0 h, the mouse was prepared for follow-up imaging of residual [^18^F]F-FDG activity. The mice were brought back to their cages and left in room-air. At 24 h after O_3_ exposure, the mice were injected with another intravenous dose of 2–5 MBq of [^18^F]F-FDG and imaged (O_3_-24 h). After imaging, mice were returned to their cages. After 4 h, i.e. O_3_-28 h, the mice were imaged again for residual [^18^F]F-FDG activity.

Three pre-weighed mice were acclimatized to the cyclotron facility, overnight, and fasted for at least 4 h before imaging. The experiment design is shown in the schematic (Fig. 1).

Mice were imaged individually. At the beginning of the experiment, mice were prepared for baseline (i.e. before O_3_ exposure) imaging. Immediately after inducing isoflurane (5%) anesthesia, mice were maintained under 1.5–2.5% isoflurane on a warm water circulated heating pad and monitored for vital signs (*BioVet*, breathing rate 40–100 breaths per min, body temperature 36.5–38 °C and blood oxygen saturation by pulse oximetry i.e. SpO_2_ 98–100%). Mice were injected with 2–5 MBq of [^18^F]F-FDG via a tail vein. Within 15–30 min post injection, mice were imaged for 3–5 full-body time-frames (800 µm voxel size), where each frame spanned 5 min (*VECTor*^4^*CT, MI Labs*). Thus, for every time-point, mice were imaged for over 25 min. [^18^F]F-FDG microPET imaging was followed by a 2 min X-ray CT (2 bed positions). After baseline imaging, the mice were continuously exposed for 2 h at 0.05 ppm O_3_ as explained in Fig. 1. Immediately after O_3_ exposure (i.e. 0 h post-O_3_ exposure), the mice underwent similar full-body microPET/CT imaging. The next day, at 24 h after O_3_ exposure, the mice were again injected i.v. with 2–5 MBq of [^18^F]F-FDG and imaged for 3–5 full body [^18^F]F-FDG PET-CT scans. Four hours later, i.e. at 28 h post-O_3_ exposure, mice were imaged for any residual [^18^F]F-FDG activity with similar full-body [^18^F]F-FDG scans.

### Image processing and analysis

**Figure 2:**
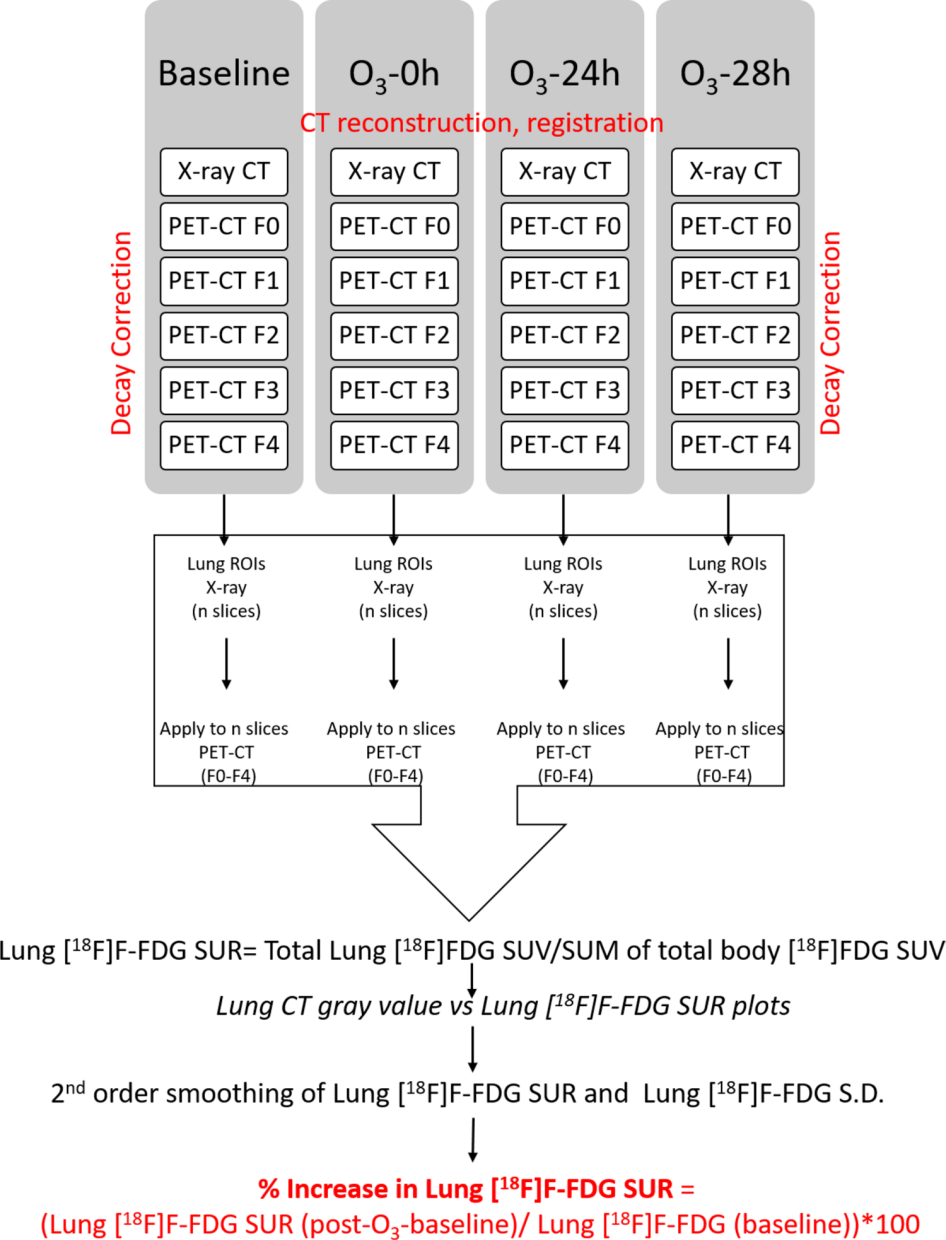
Image processing and analysis for sequential lung [^18^F]F-FDG SUR determination: The acquired X-ray and PET-CT image data sets were processed for flat and dark current normalization, reconstruction, co-registration (MI Labs software) and preliminary quantification by Pmod (pmod.com). As all the time-points, before and after O_3_ exposure, were acquired through the same imaging protocol, the [^18^F]F-FDG counts were decay corrected in order to plot tissue [^18^F]F-FDG uptake or elimination, and not decay, for the 30 min imaging time. Thereafter, the image sequences were quantified and analyzed on Image J (https://fiji.sc/#). The image stacks from X-ray CT were threshold-selected to segment out the lungs. The selected lung regions of interest (ROIs) were then copied on to the corresponding [^18^F]F-FDG PET image slices across multiple frames (F0–F4). The ROIs, from X-ray CT as well as the PET images, were simultaneously quantified for the various image parameters such as the area, perimeter, mean, median, mode, standard deviation (S.D.), range and integrated counts. After exporting the data to excel file (https://doi.org/10.6084/m9.figshare.12233576), data was sorted, filtered and analyzed for corresponding mouse time-points, frames and/or CT parameters. Finally, a SUM of the same full-body parameters of Z-stacks were analyzed for every frame in order to calculate the [^18^F]F-FDG SUV. After [^18^F]F-FDG SUV is quantified for lung slice(s) as well as the full-body, the ratio of these values, termed as the Standard Uptake Ratio (SUR), is computed. The lung CT grey values were plotted against “lung [^18^F]F-FDG SUR” or “lung [^18^F]F-FDG S.D.” which was overlaid for multiple frames on the same graph. These plots were then smoothened using the second polynomial function (Savistsky-Golay), in order to compute trends in final lung [^18^F]F-FDG SUR or S.D. and subsequently, % increase in lung [^18^F]F-FDG post-O_3_ exposure (10.6084/m9.figshare.12233576).

The acquired X-ray and PET-CT image data sets were processed for flat and dark current normalization, reconstruction, co-registration (MI Labs software) and quantification by Pmod (pmod.com). As all the time-points, before and after O_3_ exposure, were acquired through the same imaging protocol, the [^18^F]F-FDG counts were decay corrected in order to plot tissue [^18^F]F-FDG uptake or elimination, and not decay, for the 25–30 min imaging time. Thereafter, the images were quantified and analyzed on Image J (https://fiji.sc/#). The image stacks from X-ray CT were threshold-selected to segment out the lungs and confirmed with the PET-CT stacks. Depending upon the data-set, anywhere from 130 to 160 ortho slices spanned the entire lung region. The selected regions of interest (ROIs) were then copied on to the corresponding [^18^F]F-FDG PET image slices across multiple frames (F0-F4). The ROIs, from X-ray CT as well as the PET images, were simultaneously quantified for the various image parameters such as the area, perimeter, mean, median, mode, standard deviation (SD), range and integrated counts. After exporting the data to excel file, data was sorted, filtered and analyzed for corresponding imaging time-points, frames and/or CT parameters (https://doi.org/10.6084/m9.figshare.12233576). Finally, a SUM of the full-body parameters of Z-stacks were analyzed for every frame in order to calculate the full body [^18^F]F-FDG SUV (Standard Uptake Value). After [^18^F]F-FDG SUV is quantified for lung slice(s) as well as the full-body, the ratio of these values, termed as the Standard Uptake Ratio (SUR), is computed. The lung CT grey values were plotted against “lung [^18^F]F-FDG SUR”, which was overlaid for multiple frames on the same graph. These plots were then smoothened using the second polynomial function (Savistsky-Golay), in order to compute trends in lung [^18^F]F-FDG SUR or S.D. and subsequently, % increase in lung [^18^F]F-FDG post-O_3_ exposure. Please refer to Fig. 2 for flow-chart of the image processing. To investigate the [^18^F]F-FDG activity in other organs, such as the heart (representing the circulating blood pool) and urinary bladder (representing the excretory pool), we also plotted [^18^F]F-FDG time-activity curves in these organs over 25–30 min at baseline, 0, 24 and 28 h time-points.

### Ethics declarations

The current animal study design was approved by the University of Saskatchewan’s Animal Research Ethics Board (AUP 20,170,016) and adhered to the Canadian Council on Animal Care guidelines for humane animal use.

### Consent to participate/Consent to publish

Not applicable.

## Results

**Figure 3:**
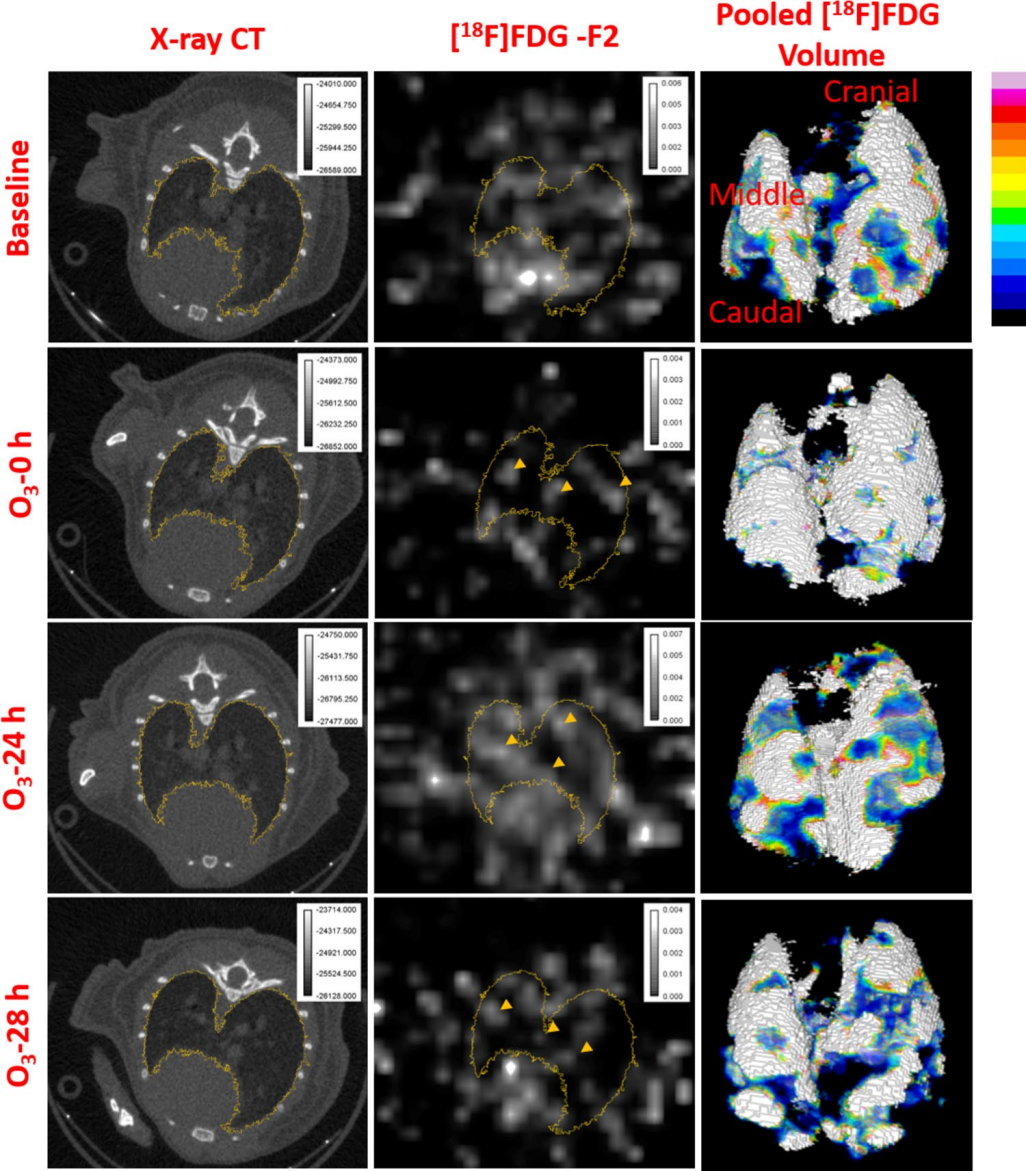
Sequential lung [^18^F]F-FDG distribution using X-ray region-of-interest: representative lung X-ray CT slice, FDG slice in the third frame (F2) and a 3-d render of the merged lung [^18^F]F-FDG volume at baseline, O_3_-0, 24 and 28 h. The 3-d render shows [^18^F]F-FDG activity normalized to16-color scale. The yellow arrow-heads indicate regions of high lung [^18^F]F-FDG uptake.

**Figure 4:**
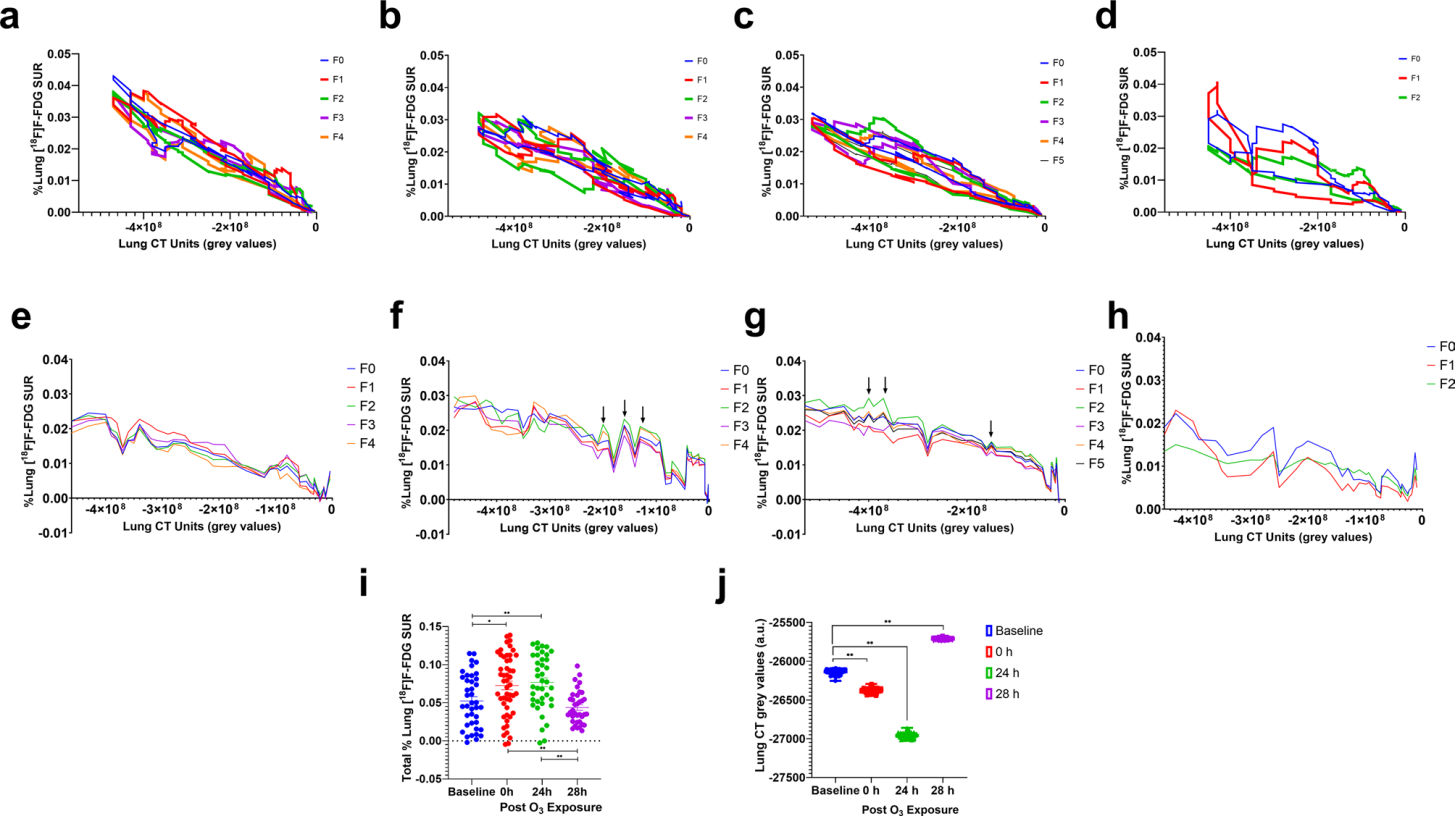
Quantification of lung [^18^F]F-FDG standard uptake ratio (SUR) and CT parameters: representative SUR data from a mouse quantified for each 5 min imaging time-frame (**a**–**d**) Percent (%) lung [^18^F]F-FDG SUR plotted against corresponding lung X-ray CT units (in arbitrary grey values), (**e**–**h**) Smoothened % lung [^18^F]F-FDG SUR plotted against the lung X-ray CT units (grey values), (**i**) three imaging time-frames were then pooled to plot the % Lung [^18^F]F-FDG at baseline, as well as 0, 24 and 28 h post-O_3_ exposure. (**j**) Regional lung mean, upper and lower range CT grey (arbitrary) values shown at baseline, as well as 0, 24 and 28 h post-O_3_ exposure. Baseline data is shown in blue, O_3_-0 h in red, O_3_-24 h in green and O_3_-28 h in purple scatter plots. Data from figures (**i**) and (**j**) were analysed by one-way ANOVA, * represents significance at *p* < 0.05 and ** at *p* < 0.01.

At baseline (i.e. within 15–30 min after [^18^F]F-FDG injection), 1.90–2.28% of [^18^F]F-FDG is extracted by lungs (Fig. 3, Movie 1) compared to the full-body uptake (Fig. 4a), with majority excreted through the kidneys (Suppl. Fig. 1). Notice the steady uptake in bladder (up to 37 min of imaging) versus already peaked concentrations in lungs (by 17 min) and heart (by 24 min) (Supp. Figs. 1a, a1).

The physical half-life of [^18^F]F-FDG decay is 109.8 min and biological half-life of 16 min, which means that > 50% of [^18^F]F-FDG was decayed by the 2nd imaging time, which is immediately after O_3_ exposure (at 0 h i.e. 2 h after baseline imaging). However, because we took account of full-body counts at every imaging time-point, regional lung [^18^F]F-FDG SUR was expressed as a percent (%) normalized lung [^18^F]F-FDG, which is irrespective of the time of imaging. Lung [^18^F]F-FDG SUR (units) varied from 0.019 to 0.0228, at baseline. The % regional lung [^18^F]F-FDG SUR varied from 7.42 × 10^−5^ to 0.043%, at baseline. Immediately after O_3_ exposure (O_3_-0 h), mid-level lung slice shows large alveolar regions devoid of [^18^F]F-FDG uptake (Fig. 3). The [^18^F]F-FDG SUV (units) varied from 0.0147 to 0.0164, at 0 h. The % regional lung [^18^F]F-FDG SUR varied from 7.42 × 10^−6^ to 0.032%, at 0 h. At 0 h, the [^18^F]F-FDG elimination kinetics is slowed in the lungs, compared to the heart and bladder (Suppl. Figs. 1a, a2). The [^18^F]F-FDG signal has a lower background signal at this time-point owing to significant renal clearance of [^18^F]F-FDG.

It must be noted that O_3_ induces instant lung tissue damage, as indicated by attenuated lung CT grey values, at 0 and 24 h post-exposure, compared to baseline values (Fig. 4j). Despite the loss of lung tissue, O_3_ induced a 72.21 ± 0.79% increase (*p* < 0.05) in lung [^18^F]F-FDG at 0 h compared to baseline (Fig. 4a, b, e, f, i). This is not very obvious in the single ortho-slice shown but evident in the volume rendered [^18^F]F-FDG uptake in lungs shown in Fig. 3 and Movie 2. Upon re-administration of [^18^F]F-FDG at 24 h post O_3_ exposure, all three organs continued to accumulate the [^18^F]F-FDG compared to the uptake phase observed in lungs and heart at baseline (Suppl. Figs. 1b, b1). The FDG SUV (units) varied from 0.0123 to 0.0166, at 24 h. The % regional lung [^18^F]F-FDG SUR varied from 1.51 × 10^−4^ to 0.032%, at 24 h. At 24 h post-O_3_ exposure, [^18^F]F-FDG was 42.54 ± 0.33% higher (*p* < 0.01) compared to baseline (Figs. 3, 4c, g, Movie 3). There were multiple areas of high concentration near the higher attenuating lung regions. The [^18^F]F-FDG SUV (units) varied from 0.0158 to 0.0183, at 28 h. The % regional lung [^18^F]F-FDG SUR varied from 3.64 × 10^−5^ to 0.041%, at 28 h. Thus, at 28 h post-O_3_ exposure, there wasn’t a significant change (*p* > 0.05) in lung [^18^F]F-FDG compared to baseline (Figs. 3, 4d, h, Movie 4), but the pattern of [^18^F]FDG uptake shifted towards the lower attenuating lung regions. The lung CT grey values also showed higher values, indicating inflammatory influx, at 28 h post-O_3_ exposure.

**Figure 5:**
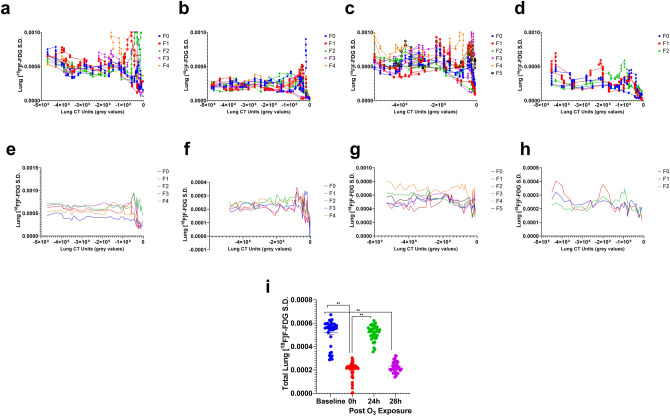
Quantification of lung [^18^F]F-FDG Standard deviation (S.D.) parameters: Representative S.D. data from a mouse quantified for each 5 min imaging time-frame (**a**–**d**) Percent (%) lung [^18^F]F-FDG S.D. plotted against corresponding lung X-ray CT units (in arbitrary grey values), (**e**–**h**) Smoothened % lung [^18^F]F-FDG S.D. plotted against the lung X-ray CT units (grey values). Please note that F0 is shown in blue, F1 in red, F2 in green, F3 in purple and F4 is shown in orange data points and line plots. (**i**) Three image time-frames were then pooled to plot the Lung [^18^F]F-FDG S.D. at baseline, as well as 0, 24 and 28 h post-O_3_ exposure. Baseline data is shown in blue, O_3_-0 h in red, O_3_-24 h in green and O_3_-28 h in purple scatter plots. Data from figure (**i**) was analysed by one-way ANOVA, * represents significance at *p* < 0.05 and ** at *p* < 0.01.

The regional lung [^18^F]F-FDG distribution standard deviation (S.D.) values indicate the variation in [^18^F]F-FDG uptake across the analysed lung regions i.e. low vs high grey value regions. At baseline, the [^18^F]F-FDG S.D. ranged from 0.000153 to 0.000958 (Fig. 5a).

We initially observed an attenuation in the lung [^18^F]F-FDG S.D. (1.78 × 10^−5^ to 3.62 × 10^−5^) at 0 h (*p* < 0.01) (Fig. 5a, b, e, f, i) but at 24 h, we observed a heterogeneous distribution (higher [^18^F]F-FDG S.D. ranging from 2.65 × 10^−4^ to 8.07 × 10^−4^, *p* < 0.01) when compared to baseline (Figs. 3, 5a, c, e, g, i). The lower S.D. at 0 and 28 h post O_3_ exposures (i.e. almost 3 h after respective [^18^F]F-FDG injections on 1st and 2nd day) are very likely due to clearance of [^18^F]F-FDG resulting in lower background activity. At 28 h post O_3_ exposure, lung [^18^F]F-FDG S.D. distribution was not significantly high (ranging from 1.12 × 10^−4^ to 4.04 × 10^−4^, *p* > 0.05), when compared to the baseline S.D. (Figs. 3, 5a, d, e, h, i). However, there were specific areas of high [^18^F]F-FDG uptake (Fig. 3). At 28 h post O_3_ exposure, lungs but not the heart, cleared a significant proportion of [^18^F]F-FDG, as observed in the time-activity curve (Suppl. Figs. 1b, b2) as well as the 3-d rendered views (Fig. 3 and Movie 4).

## Discussion

Positron emission tomographic (PET) imaging with [^18^F]F-FDG is a promising technique that may serve as a more sensitive outcome measure for pulmonary inflammation. The advantages of PET imaging include its noninvasiveness, ease of quantification, and ability to assess the entire lung. [^18^F]F-FDG-PET exploits the “Warburg effect”, the observation that many cancers use aerobic cytoplasmic glycolysis as opposed to mitochondrial glucose oxidation as a major energy source, a process that requires increased cellular glucose uptake. Aerobic glycolysis is also a characteristic of nonmalignant proliferating cells and is observed in acute lung inflammation^6,7^. Evidence to date suggests that neutrophils contribute primarily to the increased uptake of [^18^F]F-FDG in lung inflammation and that the [^18^F]F-FDG-PET signal correlates with the presence of activated neutrophils^8^. Clinical studies have also demonstrated that [^18^F]F-FDG-PET imaging can be used to assess the neutrophilic inflammatory burden in the lungs in cystic fibrosis, pneumonia, and experimentally induced lung inflammation^8–11^. These results together indicate that [^18^F]F-FDG-PET imaging can potentially be used to measure changes in pulmonary inflammation in response to anti-inflammatory treatments.

We set out to explore 2 questions: (1) the utility of dynamic [^18^F]F-FDG-PET acquisition in discriminating before and after O_3_ exposure; (2) the feasibility of [^18^F]F-FDG-PET in tracking the pathology of O_3_ induced lung inflammation in in vivo O_3_ models and their response over time (0, 24 and 28 h). Recently, Hofheinz et al. have shown that tumor to blood SUR, and not SUV, correlate with Patlak based K_m_ i.e. metabolic [^18^F]F-FDG uptake in tumors, when imaged at dual-time points^12^. Along similar lines, we performed two dual-time point imagings on the same animal to create lung [^18^F]F-FDG SUR maps, which allows longitudinal characterization of lung inflammation. Our data demonstrate time-dependent variability in [^18^F]F-FDG uptake in O_3_ exposed lungs. Dual-time [^18^F]F-FDG imaging has been done in the past to evaluate pulmonary fibrosis^13^ and idiopathic interstitial pneumonia^14^, where positive lung [^18^F]F-FDG retention is predictive of earlier deterioration of pulmonary function and mortality. However, these studies quantified [^18^F]F-FDG in a few 2–5 ROIs in lungs, whereas our study evaluates whole lung [^18^F]F-FDG distribution and provides both fractional as well as total lung [^18^F]F-FDG distribution over time. We observed a differential pattern of lung [^18^F]F-FDG distribution immediately after O_3_ exposure. The immediate (0 h) increase in O_3_-induced lung [^18^F]F-FDG was detectable as multiple spikes in the regions with lowest CT attenuation number near cranial and caudal regions. The increase in lung [^18^F]F-FDG uptake was observed despite O_3_ induced lung damage, as indicated by lowered CT values, at 0 and 24 h post O_3_ exposure. Some ortho-slices showed large gaps in [^18^F]F-FDG distribution, which also indicate the immediate alveolar damage by O_3_. Thus, the strength of our systematic regional as well as gross lung [^18^F]F-FDG analysis lies in the fact that our analysis could detect O_3_-induced metabolic activation of different alveolar regions, as a result of inflammation, despite alveolar damage. At 24 h after O_3_ exposure, the opposite pattern was observed i.e. the [^18^F]F-FDG spikes were smaller and observed in the high attenuation regions or the mid-lung regions. At 28 h after O_3_ exposure, there was an overall reduction in the lung [^18^F]F-FDG uptake. However, the distribution pattern was homogenous, indicating that the pulmonary circulation homogenously extracts [^18^F]F-FDG.

We did not report the uptake or elimination [^18^F]F-FDG rate constants in our current study, owing to fewer data points, spanning a total of 30–35 min imaging in 5 min increments, which adds to the lack of time resolution for effective uptake and elimination rate constant calculations. Future studies will aim at modifying the acquisition parameters for plotting [^18^F]F-FDG time activity curves and repeated blood sampling. Our sequential [^18^F]F-FDG imaging strategy is capable of objective analysis of the lung [^18^F]F-FDG retention before as well as up to 28 h after O_3_ exposure.

## Conclusion

Thus, our study reveals that longitudinal [^18^F]F-FDG-PET imaging may offer a tool for deep phenotyping of lung inflammation to understand the response to new targeted treatments in animal models and later in clinical trials.

## Supplementary information

Supplementary Legends.

Supplementary Fig. 1.

Supplementary Movie 1.

Supplementary Movie 2.

Supplementary Movie 3.

Supplementary Movie 4.

## Data Availability

The datasets used and/or analyzed during the current study are available from the corresponding author on reasonable request.
