# Supplementary material for: Quantification of regional murine ozone-induced lung inflammation using [18F]F-FDG microPET/CT imaging
Source: Sci Rep. 2020 Sep 24;10:15699. doi: 10.1038/s41598-020-72832-8 (PMC7515916; doi:10.1038/s41598-020-72832-8)
Supplement: Supplementary file 1 — Supplementary Legends. [file 41598_2020_72832_MOESM1_ESM.docx]

Supplementary Material

Quantification of Regional Murine Ozone-Induced Lung Inflammation Using [^18^F]F-FDG MicroPET/CT Imaging

**G Aulakh^1*^, M Kaur^1^, V Brown^1^, S Ekanayake^1^, B Khan^2^ and H Fonge^2,3^**

^1^ Western College of Veterinary Medicine, University of Saskatchewan, Saskatoon, Canada

^2^ College of Medicine, University of Saskatchewan, Saskatoon, Canada

^3^Department of Medical Imaging, RUH Saskatoon, Saskatoon, Canada

**^*^Corresponding author:** gurpreet.aulakh@usask.ca; **ORCID ID:** 0000-0002-0106-2540

**Supplementary Figures and Movies**

**Suppl. Fig. 1:** [^18^F]F-FDG time activity curves for lungs (shown in blue), heart (shown in red) and urinary bladder (shown in green) on **a)** quantified for Day 1 (over 4 h), showing **a1)** initial [^18^F]F-FDG uptake phase at baseline and **a2)** [^18^F]F-FDG elimination phase at 0 h post O_3_ exposure time-points (spanning 0.6 h). Similar time activity curves are also shown for **b)** Day 2 (over 4 h), further showing **b1)** [^18^F]F-FDG uptake phase at 24 h and **b2)** [^18^F]F-FDG elimination phase at 28 h post O_3_ exposure time-points (spanning 0.6 h).

**Movie 1:** Representative 3-d volumetric rendering of the segmented lung [^18^F]F-FDG volume at baseline. The 3-d render shows [^18^F]F-FDG activity normalized to16-color scale.

**Movie 2:** Representative 3-d volumetric rendering of the segmented lung [^18^F]F-FDG volume at 0 h after O_3_ exposure (0.05 ppm for 2 h). The 3-d render shows [^18^F]F-FDG activity normalized to16-color scale.

**Movie 3:** Representative 3-d volumetric rendering of the segmented lung [^18^F]F-FDG volume at 24 h after O_3_ exposure (0.05 ppm for 2 h). The 3-d render shows [^18^F]F-FDG activity normalized to16-color scale.

**Movie 4:** Representative 3-d volumetric rendering of the segmented lung [^18^F]F-FDG volume at 28 h after O_3_ exposure (0.05 ppm for 2 h). The 3-d render shows [^18^F]F-FDG activity normalized to16-color scale.
