# Supplementary figures and images for: Quantification of regional murine ozone-induced lung inflammation using [18F]F-FDG microPET/CT imaging
Source: Sci Rep. 2020 Sep 24;10:15699. doi: 10.1038/s41598-020-72832-8 (PMC7515916; doi:10.1038/s41598-020-72832-8)

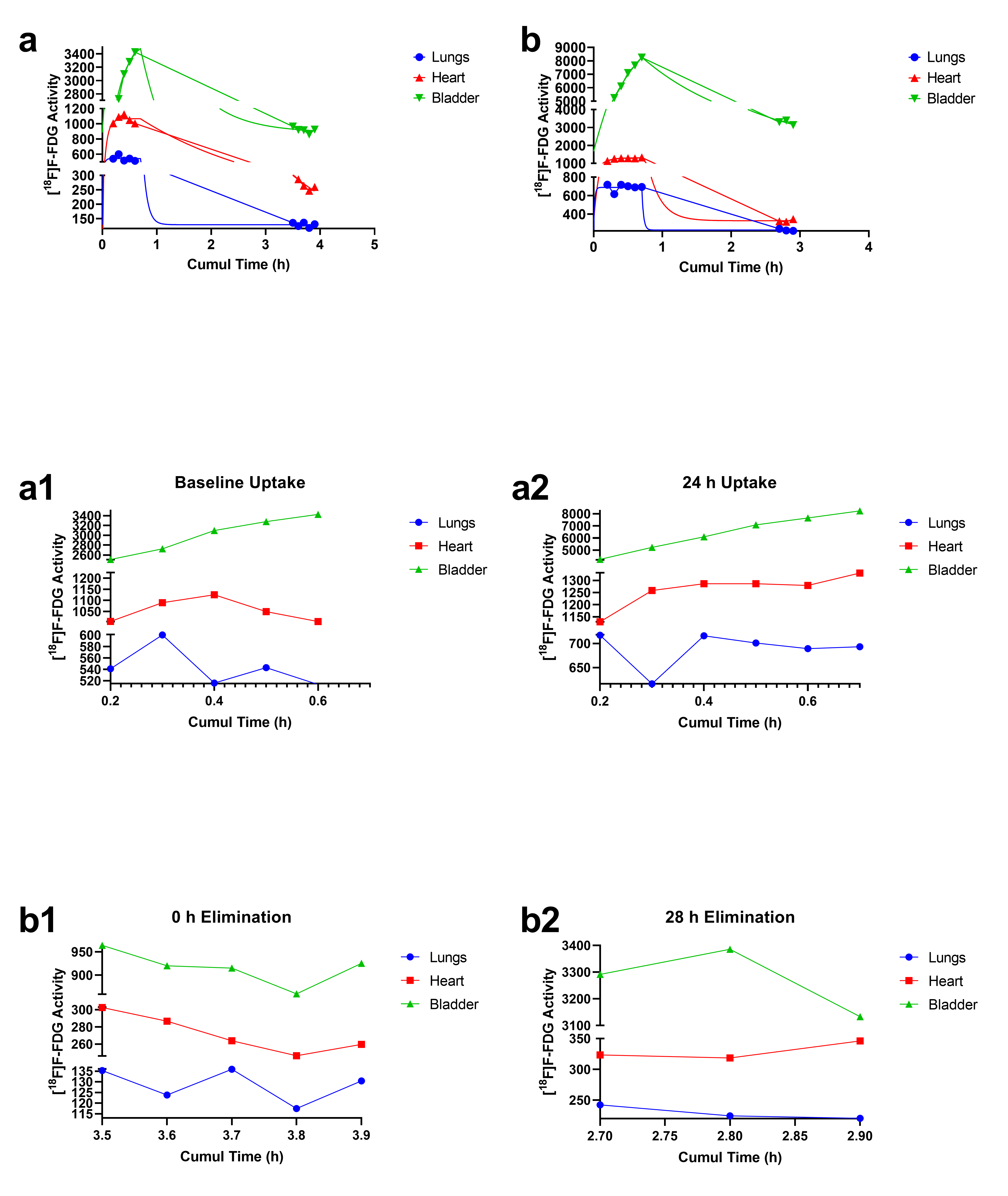

Supplement: Supplementary file 2 — Supplementary Fig. 1. [file 41598_2020_72832_MOESM2_ESM.tif]
